# A large dumbbell glossopharyngeal schwannoma involving the vagus nerve: a case report and review of the literature

**DOI:** 10.1186/1752-1947-2-334

**Published:** 2008-10-27

**Authors:** Hongyu Zhao, Xiaodong Li, Qingjie Lv, Yuhui Yuan, Hongwei Yu

**Affiliations:** 1 Department of Neurosurgery, The Second Affiliated Hospital (Shengjing Hospital), China Medical University, Shenyang, 110004, PR China; 2 Department of Pathology, The Second Affiliated Hospital (Shengjing Hospital), China Medical University, Shenyang, PR China

## Abstract

**Introduction:**

Schwannoma arising from the glossopharyngeal nerve is a rare intracranial tumor. Fewer than 40 cases have been reported. Accurate pre-operative diagnosis and optimal treatment are still difficult.

**Case presentation:**

We present one case of schwannoma originating from the ninth cranial nerve with palsies of the trigeminal nerve, facial-acoustic nerve complex, and vagus nerve in addition to ninth nerve dysfunction. Magnetic resonance imaging showed tumors located in the cerebellopontine angle with extracranial extension via the jugular foramen, with evident enhancement on post-contrast scan. Surgical management single-staged with the help of gamma knife radiosurgery achieved total removal.

**Conclusion:**

Glossopharyngeal schwannoma is devoid of clinical symptoms and neurological signs. High resolution magnetic resonance imaging may play a key role as an accurate diagnostic tool. A favorable option of approach and appropriate planning of surgical strategy should be the goal of operation for this benign tumor.

## Introduction

Intracranial schwannomas constitute approximately 8–10% of all primary brain tumors [1]. Schwannomas arising from the 9th, 10th, and 11th cranial nerves (also called jugular foramen schwannoma) without associated neurofibromatosis are relatively uncommon, and comprise only 2.9% of all intracranial schwannomas [2]. In extremely rare instances, they arise from the glossopharyngeal nerve [3]. In this report, we present one case of glossopharyngeal neurinoma and review similar cases from the literature.

## Case presentation

A 19-year-old girl came to our attention because of a history of decreased hearing and tinnitus in the right ear dating back 6 years, with a progressive swaying to the right side, accompanied by hoarseness and dysphagia for 3 months. She also complained of mild headache associated with nausea or vomiting. Neurological examination showed corneal reflex was absent on the right side. She had right side facial palsy with loss of taste. Weakness of gag reflex was present bilaterally. She had cerebellar ataxia with swaying to the right side.

**Figure 1:**
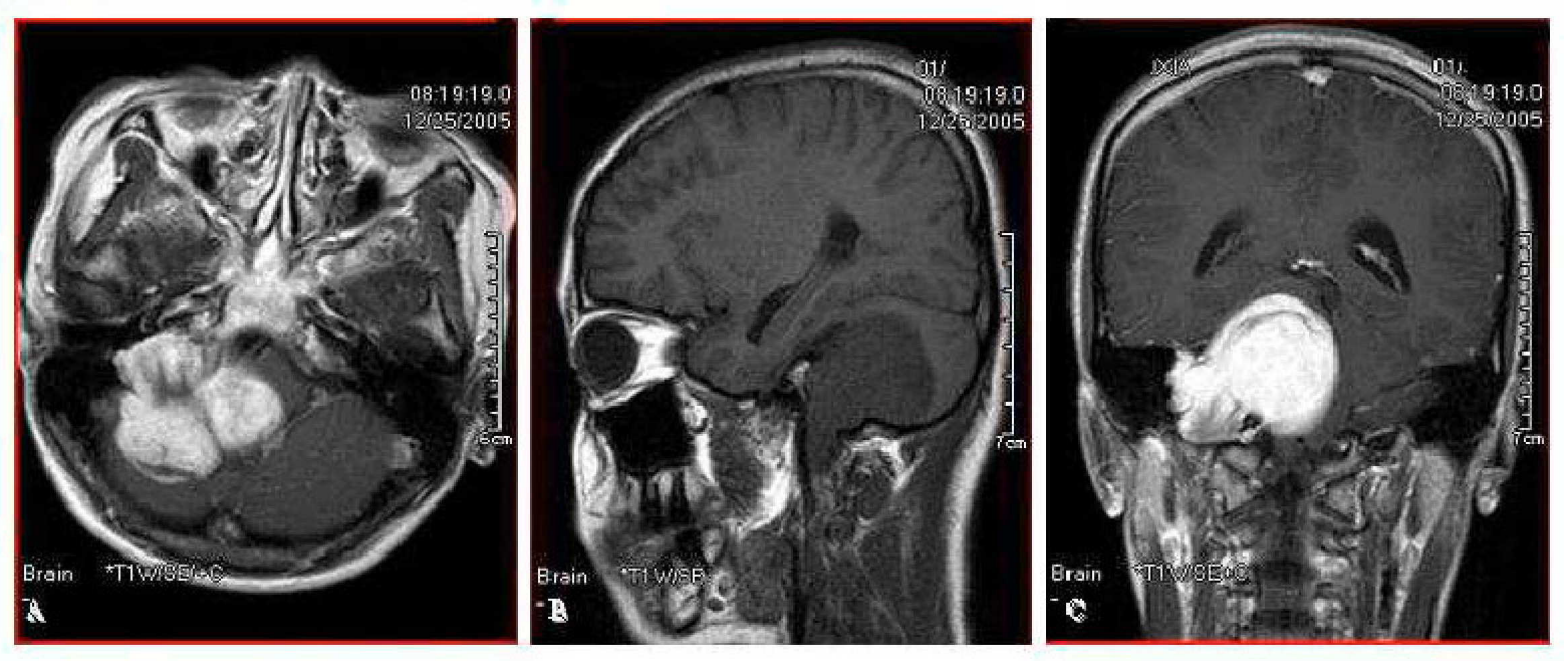
**(A) Axial gadolinium-enhanced T1-WI magnetic resonance image shows an enhanced lesion in the right cerebellopontine angle, with compression and distortion of the brain stem and cerebellum.** (B and C) Sagittal T1-WI (B) and coronal gadolinium-enhanced T1-WI magnetic resonance images (C) display a significantly enhanced mass in the right cerebellopontine angle with an extracranial extension through the jugular foramen.

**Figure 2:**
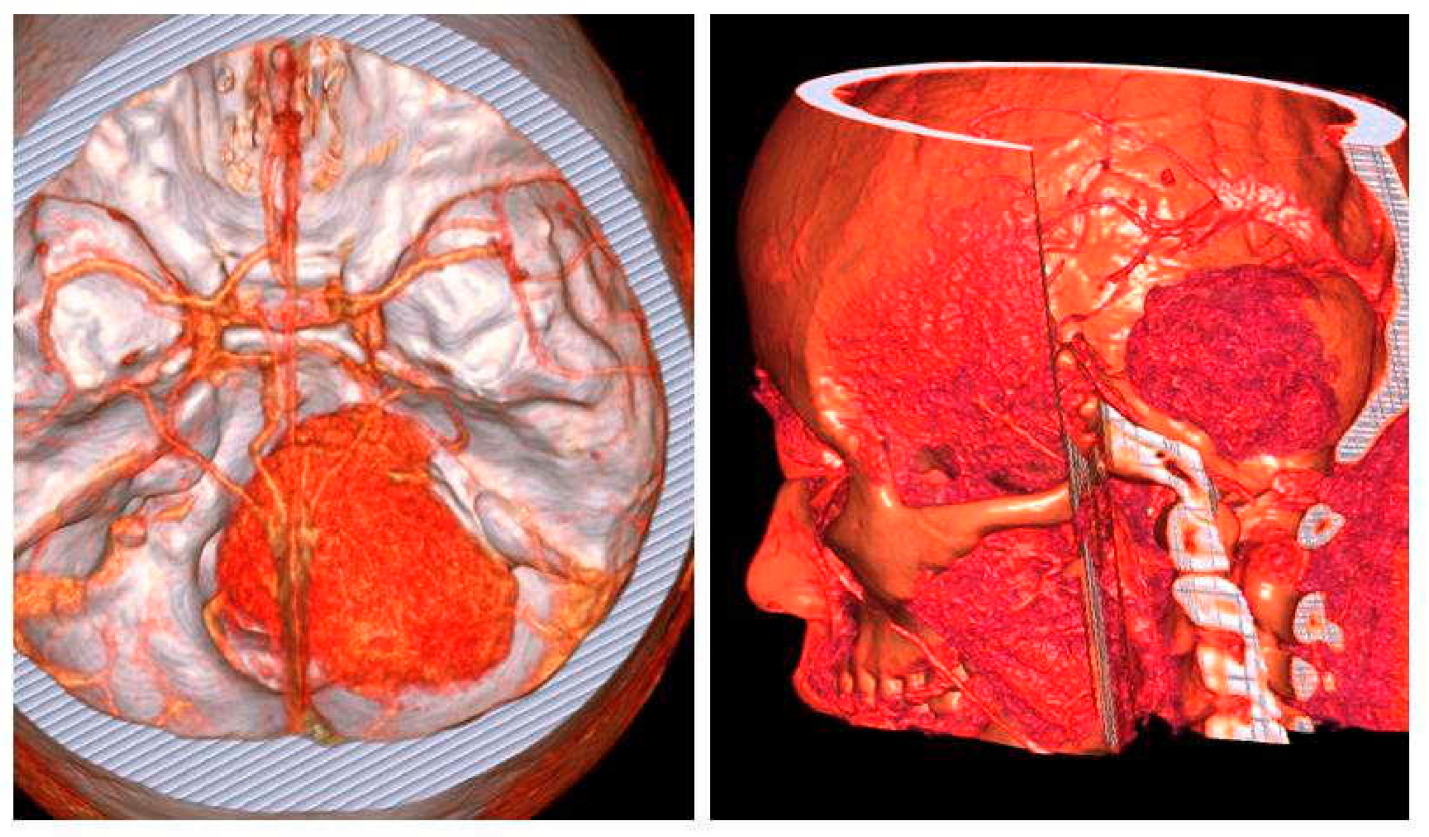
Three-dimensional computed tomography angiography image shows the status of the venous drainage system and the relationship with the regional vessels.

Magnetic resonance imaging (MRI) revealed a large solid tumor in the right cerebellopontine angle (CPA) with extracranial extension via the jugular foramen (JF). The mass was conspicuously enhanced on contrast administered T1-weighted MR image (Fig. 1). Three-dimensional computed tomography (three-dimensional CT) angiography showed the status of the venous drainage system and the relationship with the regional vessels (Fig. 2).

The patient underwent a right far-lateral transcondylar approach craniotomy in a left "park bench" position. Microsurgical piecemeal tumor debulking was performed through the CPA extending to the JF region, and the origin of the nerve was identified at surgery as the glossopharyngeal nerve. The huge mass occupied the narrow compartment so that other cranial nerves (including the VII-VIII nerve complex, X nerve, and even the V nerve) were also affected simultaneously. The presence of this large tumor with dense adhesion to the vagus nerve trunk and brainstem resulted in arrhythmia, even transient cardiac arrest during the meticulous dissection of the tenth cranial nerve from the mass. Due to this concern, it is justifiable to leave behind any tumoral capsule that is tightly adherent to the vagus nerve and brainstem and accept a subtotal removal without worsening the brainstem and vagus nerve dysfunction. Subsequently, the extracranial portion of this mass was resected *en bloc *via the JF. The osseous defect of the JF was sealed with Dura-Guard. The residual tumor was extracted with the help of gamma knife surgery after 2 weeks postoperatively.

By postoperative day 10, the girl's hearing deficit, hoarseness and dysphagia had improved greatly. A mild remaining facial palsy resolved after 6 weeks.

On histological examination, the relatively solid mass was confirmed as a schwannoma.

## Discussion

Clinical presentation of intracranial schwannoma is usually characterized by local cranial nerve dysfunction. However, since the posterior fossa is a small compartment, multiple cranial nerves may be affected simultaneously. Palsies of the ninth cranial nerve are unusual and symptoms of ninth nerve dysfunction may not become apparent until there is bilateral involvement. Furthermore, this neurinoma usually grows toward the CPA and initially affects the facial-acoustic nerve complex. Therefore, hearing loss is the most common symptom in 90–93% of cases. Hoarseness and decreased gag reflex ranked next in the review of the literature [4,5].

The radiological findings of this tumor are fairly typical, but not characteristic. MRI demonstrated soft tissue details, vascular supply of the tumor, and the relationship to the surrounding nerve structures. To date, three-dimensional CT angiography can help the clinician define the status of the venous drainage system and the relationship with the vessels near the tumor, and observe skull structure, which is of benefit in the diagnosis and surgical planning of this tumor [6]. Despite its accuracy, neuroimaging is not diagnostic of a ninth nerve schwannoma. The diagnosis is usually made when the tumor arising from the ninth nerve is seen at surgery. In our case, an accurate pre-operative diagnosis of lower cranial nerve schwannoma was made based on clinical presentation and radiological appearance.

The surgical approach to remove glossopharyngeal schwannomas should be selected according to the location and degree of extension of the individual tumor. Sammi et al. classified these tumors into Type A, B, C, and D according to the radiological and surgical features [2]. The complex anatomy of the skull base around the JF makes total removal of these tumors, especially Type D tumor, technically difficult. In this patient, the dumbbell shaped tumor was identified as Type D. A far-lateral transcondylar approach was used by which this huge tumor with both intra- and extracranial extension could be subjected to single-stage removal. We feel that this choice of route gives several advantages, including: 1) giving the facial-acoustic nerve, lower cranial nerves and neighboring major blood vessels a good exposure; 2) providing direct access to the jugular foramen and neck; and 3) in case of injury to the spinal accessory nerve during the cervical portion of tumor resection, skull base reconstruction can be performed easily. The dissection of the tumoral capsule from the lower cranial nerves is the most difficult and challenging step in the entire course. To lessen lower cranial nerve deficits, it is acceptable to leave behind a tumoral capsule that is intensively adherent to the cranial nerve trunks and brainstem. In addition, cerebrospinal fluid (CSF) leakage is also a formidable complication after total resection of such a large tumor [7]. In this patient, a partial resection of this huge mass was performed because of the risk of damaging the vagus trunk and brainstem. Currently, stereotactic radiosurgery and gamma knife radiosurgery can provide further treatment for those patients who have a residual tumor after their surgical resection [8]. This patient underwent gamma knife treatment 2 weeks postoperatively. The residual tumor adherent to the tenth nerve and brainstem disappeared completely without new cranial nerve deficit and other complications. In the future, a navigation-aided procedure may enable greater technical precision by tracking the surgical trajectory while also displaying the tumor's location on neuronavigation images.

## Conclusion

The differential diagnosis of glossopharyngeal schwannoma is still very difficult, because specific clinical symptoms and radiological findings can be absent in most cases. With careful, extensive pre-operative evaluation and appropriate planning of the surgical approach, as well as using innovative therapeutic strategies, glossopharyngeal schwannoma can be radically and safely resected without creating additional neurological deficits and other complications. Furthermore, recovery of cranial nerve dysfunction can be expected.

## Consent

Written informed consent was obtained from the patient for publication of this case report and any accompanying images. A copy of the written consent is available for review by the Editor-in-Chief of this journal.

## Competing interests

The authors declare that they have no competing interests.

## Authors' contributions

HZ performed the literature review on similar cases, and wrote the manuscript. XL collected the patient's data while QL performed the histological examination of the tumor. YY and HY gave final approval of the version to be submitted for publication. All authors read and approved the final manuscript.
